# Hepatitis E virus infections in German blood donors: results of 8 years of screening, 2015 to 2022

**DOI:** 10.2807/1560-7917.ES.2024.29.24.2300665

**Published:** 2024-06-13

**Authors:** Ricarda Plümers, Jens Dreier, Cornelius Knabbe, André Gömer, Eike Steinmann, Daniel Todt, Tanja Vollmer

**Affiliations:** 1 Institut für Laboratoriums- und Transfusionsmedizin, Herz- und Diabeteszentrum Nordrhein-Westfalen, Bad Oeynhausen, Germany; 2 Department for Molecular und Medical Medicine, Ruhr University Bochum, Germany; 3 German Centre for Infection Research (DZIF), External Partner Site, Bochum, Germany; 4 European Virus Bioinformatics Center (EVBC), Jena, Germany

**Keywords:** Hepatitis E, blood donor, blood safety, NAT testing, seroprevalence, epidemiology

## Abstract

**Background:**

Awareness of transfusion-transmitted hepatitis E raised in recent years led to the mandatory testing of blood donations in some European countries for hepatitis E virus (HEV) RNA. However, little is known about the epidemiology of HEV infections.

**Aim:**

To and describe and analyse the epidemiology of HEV infections in blood donors in Germany.

**Methods:**

Data from routine testing of therapeutic blood products donated between January 2015 and December 2022 at the Uni.Blutspendedienst OWL were analysed at the Institute of Laboratory and Transfusion Medicine, Heart and Diabetes Centre North Rhine-Westphalia. A total of 731,630 allogenic blood donations from 119,610 individual blood donors were tested for HEV RNA in minipools of 96 samples. The HEV RNA-positive donations were analysed for the presence of anti-HEV IgM and IgG. The HEV strains were genotyped and various clinical liver-specific parameters were determined.

**Results:**

A total of 497 HEV-positive blood donations were identified, resulting in a yearly incidence of 1:1,474, from which 78.4% of the donations were RNA-only positive. Increased alanine aminotransferase activity was determined in 26.6% of HEV RNA-positive donors and was associated with the detection of IgG antibodies (1.2% anti-HEV IgM-positive, 11.9% anti-HEV IgM- and IgG-positive and 8.5% anti-HEV IgG-positive). An average incidence of 0.084–0.083% HEV RNA-positive donations in June and July in all years was observed, and a higher proportion of HEV RNA-positive men compared with women. All isolated HEV sequences corresponded to genotype 3.

**Conclusion:**

Our results underline the necessity of HEV RNA screening in blood donations.

**Key public health message**

**What did you want to address in this study and why?**

Hepatitis E is a liver disease caused by the hepatitis E virus (HEV). Transmission of HEV via by blood transfusion may cause severe and chronic hepatitis in high-risk patients. Little is known about the spread and epidemiology (age, sex, season) of HEV as the infection is rarely recognised in apparently healthy people. We aimed to address this by analysing data from HEV-testing of blood products from our German blood donation facility from 2015 to 2022.

**What have we learnt from this study?**

Calculations showed an overall incidence of HEV infections of 1:1,474 blood donations. There was no evidence that age or season has any influence on the risk of HEV infection. However, while there were 71 females for every 100 males among donors overall, there were only 36 females for every 100 male HEV-positive donors showing an imbalance in susceptibility between men and women.

**What are the implications of your findings for public health?**

We can now better assess how HEV infections are distributed in the population in Germany and how high the actual risk of infection through blood transfusion is. In addition, our data show that even 26.65 % of apparently healthy donors have altered liver parameters that are associated with an advanced HEV infection, which is reflected in the formation of antibodies.

## Introduction

Hepatitis E became a notifiable disease in Germany in 2001 [1]. The number of reported persons with acute hepatitis E (symptomatic with laboratory confirmation) has increased 40-fold in the past 10 years [2]. It is currently unknown whether the increase in hepatitis E virus (HEV) infection prevalence is fuelled by more frequent transmission or by higher awareness and testing. Hepatitis E virus has received attention in recent years due to reports of chronic hepatitis E in immunosuppressed patients [3]. Another principal driver of awareness may be the extensive discussion in blood establishments worldwide on whether there is a need for general blood donor screening for HEV. The most prevalent HEV genotype in Germany and the rest of Europe is genotype 3 (HEV-3) and is usually transmitted via the zoonotic or food-borne route [4]. The transmission of HEV-3 infection via the transfusion of various blood products has been described previously [5]. A study by Westhölter et al. reporting routine HEV screening experiences concluded that HEV-positive blood donations represent a relevant infection risk for immunosuppressed recipients [6]. Studies reported incidences of HEV RNA-positive donations made in Germany between 2011 and 2018 ranging between 1:815 and 1:4,525 [6-8]. Nationwide mandatory HEV RNA screening of blood donations has been introduced in Austria, France, Ireland, Luxembourg the Netherlands, Spain and the United Kingdom (UK), and the Paul-Ehrlich Institute mandated the introduction of HEV RNA screening in Germany in January 2020 [8,9].

Studies based on the screening of blood donors for HEV RNA in Europe reported the number of unrecorded cases previously overlooked due to asymptomatic HEV infections in healthy people, with incidences ranging from 1:157 in central Italy to 1:14,520 in Scotland [10]. However, most of the studies comprised a short period of 1 year or less. Therefore, multi-year studies with large blood donor cohorts became the focus of epidemiological research. These have been conducted in England (2016–2017; incidence 1:3,830; 1,838,747 donations), Finland (2016–2022; incidence 1:5,784; 23,137 donations), France (2015–2021; incidence 1:5,784; 510,118 donations) and Spain (2017–2020; incidence 1:4341; 655,523 donations) [11-14].

As concordant data are missing for Germany, we present data on HEV infections in blood donations obtained from routine HEV screening over a period of 8 years, and tested at our facility, where we also obtained serostatus and measured liver-specific parameters. Since the observation period comprises several years, our findings contribute to the understanding of HEV epidemiology in Germany regarding age and sex and seasonal differences.

## Methods

### Blood donors and hepatitis E virus nucleic acid test screening

The HEV RNA screening of therapeutic blood products, donated at our blood donation service Uni.Blutspendedienst OWL (donors from North Rhine-Westphalia, Lower Saxony and Hesse), was introduced in January 2015. Here we analysed all donations from January 2015 to December 2022. The HEV RNA screening was performed in minipools (MP) of 96 samples. Three different assays for screening were used over time: (i) January 2015 to November 2020: RealStar HEV RT-PCR Kit (Altona Diagnostic Technologies, Hamburg, Germany), 95% limit of detection (LOD): 4.66 IU/ml (95% confidence interval (CI): 3.6−7.5 IU/ml), detection limit of 447.4 IU/ml for a single donation in a 96 sample MP [15]; (ii) January 2015 to November 2020: AltoStar HEV RNA RT-PCR Kit (Altona Diagnostic Technologies), 95% LOD: 3.41 IU/ml (95% CI: 2.28–6.4 IU/ml), detection limit of 327 IU/ml for a single donation in a 96 sample MP; (iii) November 2020 to December 2022: cobas HEV assay (Roche Diagnostics, Basel, Switzerland), 95% LOD: 18.6 IU/ml (95% CI: 15.9–22.6 IU/ml), detection limit of 1,786 IU/ml for a single donation in a 96 sample MP. The minimum sensitivity requested by the Paul-Ehrlich Institute is 2,000 IU/ml [9].

All HEV-infected donors underwent a pre-donation medical examination and fulfilled the criteria for donating blood (older than 18 years, maximum donation volume 2,000 mL for women and 3,000 mL for men per year) with no current diseases or any known risk factors for viral infection. The blood donations that tested positive during HEV RNA screening were immediately rejected for transfusion.

### RNA extraction and quantitative real-time PCR (qRT-PCR)

Total RNA sequenced from individual samples was extracted from 500 µl plasma using the NucliSens easyMAG (bioMerieux, Nürtingen, Germany) or AltoStar AM16 (Altona Diagnostic Technologies) automated RNA/DNA extraction system. Amplification using the RealStar HEV RT-PCR Kit (Altona Diagnostic Technologies) was performed on the Rotor-Gene 3000 system (Corbett Life Sciences, Sydney, Australia). Amplification using the AltoStar HEV RNA RT-PCR Kit was performed on the Bio-Rad CFX96 system (Bio-Rad, Hercules, United States (US)). Amplification using the cobas HEV assay was performed on the cobas 6000 system (Roche Diagnostics). The HEV titre in positive plasma was quantified using the first World Health Organization international standard for HEV RNA for nucleic acid test-based assays (Paul-Ehrlich Institute, Langen, Germany) [16].

### Hepatitis E virus genotyping and phylogenetic analysis

Hepatitis E virus RNA was amplified by a modified nested reverse transcription PCR in the hypervariable region (HVR) and open reading frame 1 (ORF1) region using primers according to Vina-Rodriguez et al. as described in the Supplemental Material S1 [17]. The nt sequencing was performed using the BigDye Terminator version 1.1 Cycle Sequencing kit with the 3500 Genetic Analyzer DNA sequencer (both Applied Biosystems, Foster City, US), as described previously [18]. All sequences were aligned and analysed by BioNumerics software version 6.6 (Applied Maths, Sint-Martens-Laterm, Belgium).

Sequencing data were aligned using Clustal Omega version 1.2.3 [19] and subsequently used for phylogenetic analysis. The maximum-likelihood tree was generated using IQtree2 with 1,000 bootstraps and visualised in R [20] using the following libraries: tidyverse, ggtree, treeio and phylobase.

### Serological testing and measurement of liver-specific parameters

The plasma of HEV RNA-positive donors was screened for the presence of HEV-specific IgM and IgG antibodies using anti-HEV ELISA (Euroimmun, Lübeck, Germany). Analysis and serostatus interpretation were performed according to the manufacturer’s recommendations. Follow-up samples to confirm seroconversion were analysed after a median of 142 days. Concentrations of alanine aminotransferase (ALT), aspartate aminotransferase (AST), total bilirubin (T-BIL; cutoff 0.1 mg/dL), glutamate dehydrogenase (GLDH; cutoff 2 U/L) and pseudocholinesterase (PCHE) were measured in plasma samples using the respective enzymatic assays on the Architect c8000 system (assays and system: Abbott Diagnostics Europe, Wiesbaden, Germany).

### Statistical analysis

Values are given as median with interquartile range (IQR) or 95% CI. Incidences were calculated by dividing the number of cases of HEV-positive donations by the total number of donations analysed. The cumulative incidence is based on the cumulative number of donors for the period. The cumulative number of donors includes all donors in a multi-year period and increases annually by the number of first-time donors. Significance levels were determined with nonparametric two-tailed Mann–Whitney U tests with significant results of p < 0.05. Data presentation and calculations were conducted using the GraphPad Prism 9.0 software (GraphPad Software, San Diego, US).

## Results

### Donors’ characteristics and epidemiology

A total of 731,630 allogenic blood donations (305,261 from males, 426,369 from females) corresponding to 119,610 individual German blood donors (60,243 male, 58,870 female) who donated to the Uni.Blutspendedienst OWL were screened for the presence of HEV RNA between 2015 and 2022 (1.41% of all donations made in Germany [21]). The screening recovered 497 HEV RNA-positive donations giving an incidence of 1:1,474 over the 8-year period (0.068%). Yearly incidences per donation or per donor varied over the observation period as presented in Table 1. We did not observe a steady decline or an increase in incidence over time considering the highest incidence was in 2019 (1:1,032 for donations, 1:354 for donors) and the lowest in 2021 (1:2,237 for donations and 1:924 for donors). The probability of an individual blood donor acquiring an HEV infection during a specific period depends on the calculated incidence. An incidence, for example, of 1:504 was found in 32,815 donors from 2017, whereas the cumulative data from 2015–2017 concerning a total of 56,374 donors, showed an incidence of 1:283. The cumulative incidence rose over the observed period and levelled off at an overall incidence of 1:241.

**Table 1:** Numbers of donations, donors (presented for each year isolated and cumulative over past years), hepatitis E virus RNA-positive donations, and corresponding incidences, Germany, 2015–2022

Year	Number				Incidence of HEV RNA-positive samples		
	Donations	Donors	Cumulative	HEV RNA-positive	per donation	per donor	Cumulative
2015	93,370	30,855	NA	66	1:1,414	1:468	NA
2016	97,323	32,953	44,442	68	1:1,431	1:485	1:332
2017	95,574	32,815	56,374	65	1:1,470	1:504	1:283
2018	97,577	33,560	68,654	75	1:1,301	1:447	1:251
2019	96,053	32,936	80,366	93	1:1,032	1:354	1:219
2020	94,990	35,204	94,306	56	1:1,696	1:628	1:223
2021	82,785	34,209	106,088	37	1:2,237	1:924	1:231
2022	73,958	33,083	119,610	37	1:1,998	1:894	1:241
Overall	731,630	119,610	NA	497	1:1,474	1:241	NA

HEV: hepatitis E virus; NA: not applicable.

**Figure 1:**
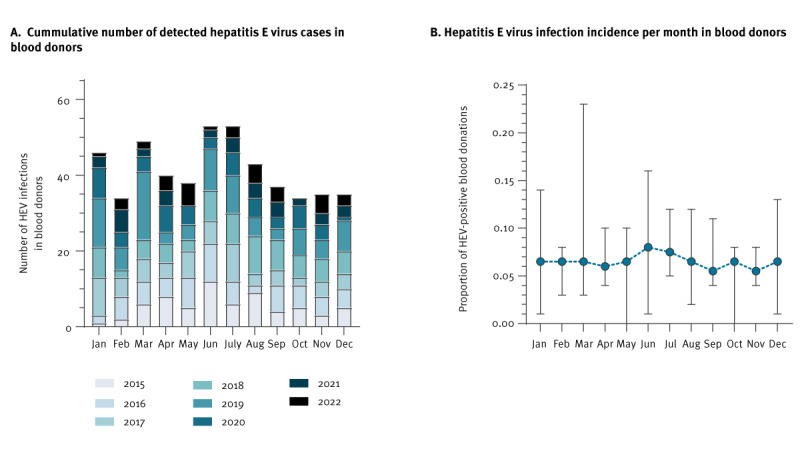
Hepatitis E virus infections in blood donors by month (A) cumulative number (B) incidence, Germany, 2015–2022

Figure 1 shows the number of HEV RNA-positive donations detected cumulatively over the years for each month and the mean incidence among blood donations over the course of a year. The highest number of HEV RNA-positive donations was identified in June and July (53 each, Figure 1A), resulting in the highest, although not significantly different, overall incidence of 0.084% (June) and 0.083% (July) (Figure 1B).

HEV: hepatitis E virus.

**Figure 2:**
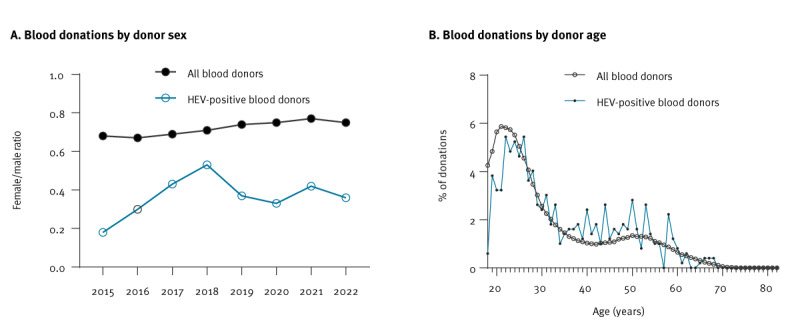
Epidemiologic characteristics of hepatitis E virus RNA-positive blood donors by (A) sex, and (B) age, Germany, 2015–2022

The ratio of female to male donors in the HEV RNA-positive cohort in comparison to the entire donor cohort is shown in Figure 2A. A value of 0.68, as determined in 2015 for the entire cohort of blood donors, means that there are 6.8 female donors for every 10 male donors. In the same year, the value of 0.18 for HEV-positive blood donors indicates that there are 1.8 female HEV-positive donors for every 10 male donors. This parameter was consistently lower in the HEV RNA-positive cohort (0.36) than in the overall donation cohort (0.71) cumulated for the study period. (Figure 2A).

HEV: hepatitis E virus.

The distribution of donations in terms of donor age showed no significant differences between the total cohort and the HEV RNA-positive cohort (Figure 2B). Furthermore, mean age did not differ significantly between the two groups (total blood donors 35.1 years ± 13.6, HEV RNA-positive donors 34.8 years ± 12.5, p = 0.3973).

### Laboratory parameters of hepatitis E virus-infected blood donors –

Serostatus was determined for 495 of the 497 HEV RNA-positive donors, of which 387 (78.4%) were negative for anti-HEV IgM and anti-HEV IgG. Six donors had a positive IgM titre only (1.2%). Fifty-nine donors showed reactive IgM and IgG titres (11.9%). The remaining 42 HEV RNA-positive donors had positive IgG titres only (8.5%).

The quantification of liver-specific parameters and viral load are summarised in Table 2 and displayed in Supplementary Figure S1.

**Table 2:** Median, percentiles and range of viral load and liver-specific parameters in samples from hepatitis E virus RNA-positive blood donors, Germany, 2015–2022 (n = 495)

	Median	25%ile	75%ile	Range	Norm values
Viral load (IU/mL)	1,660.0	342.5	11,650.0	0–1.4 × 10^7^	NA
ALT (U/L)	29.5	20.0	48.0	6.0–904.0	10–35^a^/50^b^
AST (U/L)	29.0	23.0	39.0	12.0–466.0	10–35^a^/50^b^
T-BIL (mg/dL)	0.4	0.3	0.6	0.1–2.8	0.2–1.0
GLDH (U/L)	2.7	2.0	4.9	2.0–115.0	< 5^a^/7^b^
PCHE (U/L)	9,575.0	7,960.0	10,800.0	4,280–15,700	5,859–13,060

ALT: alanine aminotransferase; AST: aspartate aminotransferase; GLDH: glutamate dehydrogenase; PCHE: pseudocholinesterase; T-BIL: total bilirubin.

^a^ Female reference value.

^b^ Male reference value.

Deviations from reference values of ALT were detected in 26.65% of HEV RNA-positive blood donors. Moreover, the liver parameters in HEV RNA-positive blood donors diverged compared with reference values in 13.0% of donors for AST, 15.0% for T-BIL, 13.1% for GLDH and 7.7% for PCHE.

**Figure 3:**
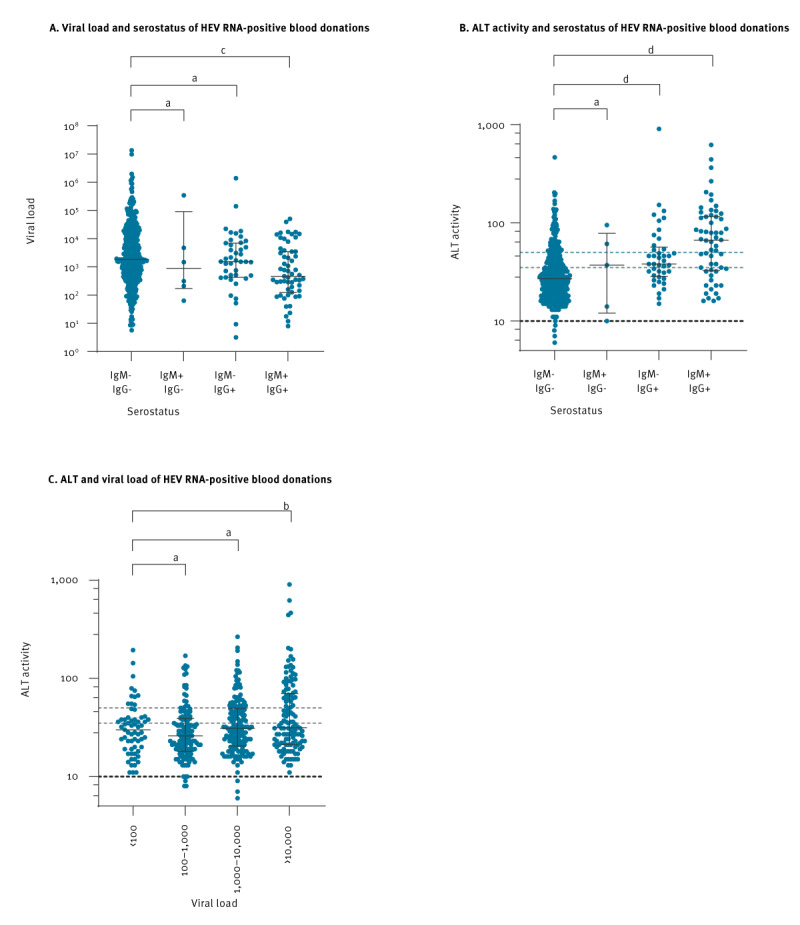
Comparison of (A) viral load and serostatus and (B) alanine aminotransferase activity (U/I) and serostatus and (C) alanine aminotransferase activity (U/I) and viral load in blood samples from hepatitis E virus RNA-positive blood donations, Germany, 2015–2022

ALT: alanine aminotransferase; HEV: hepatitis E virus.

Considering the most striking deviations of ALT, we focused on the association of this parameter with the viral load and serotype, as displayed in Figure 3.

The ALT reference values are presented as dotted lines.

Significance level of Mann–Whitney-U Test:

^a^ not significant, p > 0.05;

^b^ 0.01 < p <0.05;

^c^ p 0.001 < p < 0.01;

^d^ p < 0.0001.

Median values of viral loads in blood donors positive for either anti-HEV IgM (median 893 IU/ml, range 63.3–3.5 x 10^5^) or anti-HEV IgG (median 1,540 IU/ml, range 0–1.4 x 10^6^) did not significantly differ from the viral load in antibody-negative donations (median 1,830 IU/ml, range 0–1.4 x 10^7^). However, the median viral load in blood donations positive for anti-HEV IgM and anti-HEV IgG was significantly lower (median 466 IU/ml, range 0–5.1 x 10^4^) (Figure 3A).

In contrast, median ALT values in seronegative donors (median 27 U/L, range 6–465 U/L) were significantly lower than in donors positive for anti-HEV IgG only (median 38 U/L, range 15–904 U/L) or positive for anti-HEV IgM and anti-HEV IgG (median 66.5 U/L, range 16–621 U/L). A significant elevation of ALT activity in anti-HEV IgM positive only donors (median 37 U/L, range 10–95 U/L) was not detected possibly due to a low number of cases (n = 6) (Figure 3B).

The ALT values were not significantly different between HEV RNA-positive blood donors regarding their viral load (Figure 3C).

### Follow-up samples of hepatitis E virus infected blood donors

Confirmation of HEV infection by determining serostatus was performed for 369 returning donors on the first donation after a deferral period of 3–4 months (prerequisite HEV RNA-negative). Samples were analysed after a median of 142 days (IQR: 112–245). A total of 187 returning donors exhibited complete seroconversion with IgM-negative and IgG-positive status (50.7%). Another 158 donors were anti-HEV IgM- and IgG-positive (42.8%). Twenty-three donors remained seronegative (6.2%). One donor was anti-HEV IgM-positive only (0.3%).

### Sequence analysis of hepatitis E virus genotypes detected

We performed Sanger sequencing of the HVR and ORF1 regions to determine the HEV genotype, which was successful in 123 samples. A maximum likelihood phylogeny was then calculated to infer similarity to reference HEV strains. All samples clustered with HEV-3 reference strains and no similarity to other genotypes could be detected, as shown in Supplementary Figure S2. In addition, most sequences clustered closely with the HEV-3c reference strain (FJ70535). Furthermore, the serostatus of the patients, the location of the donor centre and the year of sampling were highlighted on the top nodes of the phylogeny. However, no common clustering pattern was observed depending on these variables as indicated in Supplementary Figure S3.

## Discussion

Our study represents a large and homogenous cohort of blood donors over a period of 8 years. We determined an overall HEV incidence of 1:1,474 in relation to total blood donations and 1:241 in relation to total donors, with an even distribution of age and season of donation and an excess of male compared to female HEV-positive donors. High viral loads and an anti-HEV IgG-positive serostatus illustrated an association with an increase in ALT activity.

A study by Tedder et al. based on data obtained in the UK determined that the risk of HEV infection from more than 13 transfusions exceeds that of infection from diet. The decisive factor in infection is the viral load, with the authors stating the lowest infectious dose of 2 x 10^4^ IU/ml, with 60% risk of infection above this dose [22]. Calculations based on Dutch data showed that the cost of treating a chronic disease is 10 times the annual cost of testing in MP of 24 samples [23]. This awareness has led to the mandatory testing of blood products for HEV RNA in several European countries, including Germany since 2020 [8].

About 24.6 million red blood cell concentrates (RBC) and 5.4 million plasma products were transfused in Germany between 2015 and 2022 (pathogen-reducing treated excluded) [21]. Assuming the HEV overall incidence of 1:1,474 from the present study, if there was no HEV screening, this would result in the administration of ca 16,600 RBCs and ca 3,700 plasma products from infected donors. Considering the residual plasma fraction of the transfused products (a mean of 7.5% for RBCs and 78.5% for plasma), a minimal infectious dose of 2 x 10^4^ IU is transfused when the original donation has a viral load above 888 IU/ml for RBCs (58.5%, ca 9,711 infectious products) and above 85 IU/ml for plasma products (88.1%, ca 3,260 infectious products) [5,16]. If we now assume that 60% of these 12,971 infectious products actually lead to infection, and 5% of the transfusion recipients infected have a severe course and 1% a chronic course, as assumed in calculations for Australia and Finland [11,24], this results in 389 cases of severe and 78 cases of chronic infections in the observed 8-year period in Germany.

The incidences observed in our study are comparable to those reported in previous publications investigating HEV infections in Germany [5,25]. All of the viruses sequenced in the present study were HEV-3, and most of them HEV-3c, as would have been expected based on global distribution considering the predominance of HEV-3 in Europe [4]. In a European comparison, Germany has a higher or comparable prevalence than, for example, the UK or the Netherlands [10]. It can thus be assumed that the risk and associated burden on the health system of transfusion-transmitted hepatitis E is comparable or even higher than in the studies mentioned above.

Incidence of HEV infection in blood donors in various other countries show considerable differences ranging from 1:157 in central Italy to 1:14,520 in Scotland [10]. On the one hand, these differences could be explained by the sensitivity of the nucleic acid test assay used for screening concurring with the MP size. We have already shown that the detection frequency of HEV RNA-positive donations was ca 50% higher using individual screening compared with MP-screening [25]. Moreover, we detected a slight decline in the number of HEV cases among blood donors in our study for the years 2020–2022 as well as a higher median viral load (2015–October 2020: 1,540 IU/mL; November 2020–2022: 1,920 IU/mL) and a lower monthly incidence (2015–October 2020: 0.07%, 95% CI: 0.06–0.08; November 2020–2023: 0.05%, 95% CI: 0.03–0.06) possibly corresponding to the lower sensitivity of the cobas HEV assay used during this period. Whether or not the decrease in sensitivity of the cobas assay compared with the AltoStar assay leads to considerable underestimation of prevalence must be clarified in a direct comparative study [22]. It can be assumed that the different sensitivities have a minor influence on the safety of blood products, as the lowest detected viral loads for both systems (AltoStar 3.2 IU/mL; cobas 11.9 IU/mL) are well below the minimum infectious viral load of 85 IU/ml in plasma products. On the other hand, varying incidences may be an effect of the donor population (dietary behaviour) and variations in HEV infection incidence over time (short-term: seasonal differences, long-term: varying disease burden). Most studies hitherto only span shorter observation periods or smaller case numbers, and the long-term observation of HEV RNA-positivity presented in this study elucidated this magnitude of influence through the combined observation of a large cohort over a long period of time [11,12,14]. Regarding HEV positivity in a seasonal comparison, our study did not find a multiple increase in incidence cumulated over years for a specific month, as has been reported for 17–24-year-old donors between March and June in the UK [12]. While the highest overall incidence in the current study occurred in June and July, we reported highly variable monthly prevalence due to the small number of cases included. A case-control study on a German cohort carried out between 2012 and 2014 has raised issues on the consumption of undercooked pork or wild boar meat as a risk factor for infection [26]. We can only speculate that the consistently high incidence in the summer season might be due to the barbecue season. We are, therefore, currently evaluating a case-control study considering seasonal issues.

A decrease in anti-HEV IgG seroprevalence between 1996 and 2011 has been discussed by Wenzel et al., but this was countered by an increase in seroprevalence, particularly in younger people, in the data of Zaaijer [27,28]. Our study enabled us to assess the infection situation directly over time without detecting a sustained decline or increase in prevalence between 2015 and 2022.

A comparison of European studies shows differences in the distribution of HEV RNA-positive donors across age groups. An Irish study on a cohort with a median age of 37 years described the highest prevalence of HEV-positive donors in the 18–24 years age group, while the majority of HEV RNA-positive donors in a Dutch study were between 51 and 60 years old [8]. A study in Scotland showed the highest seroprevalence in blood donors younger than 44 years, although the median age of all donors was 47 years [29]. Our data comparing the age groups in the total donor cohort and the HEV RNA-positive cohort showed no significant difference between the groups, either in overall distribution or in median age.

Statements on biological sex-specific differences in HEV research refer mostly to seroprevalence and show no significant differences between males and females, for example in the study by Farber et al. on a German cohort [26]. However, we found a significant proportional excess of male HEV RNA-positive donors compared with the total donor population in our data that exceeded the usual bias of a male surplus in blood donation. Interestingly, a study in Bulgaria showed that although the prevalence of past HEV infections (IgG-positive, IgM-negative, HEV RNA-negative) was similar in males and females, the prevalence of recent or ongoing infection (IgM- or HEV RNA-positive) was twice as high in men [30]. Future studies on the course of seroconversion in males and females may provide deeper insights into this phenomenon.

Regarding the analysis of the liver-specific parameters, the HEV RNA-positive cohort deviated from the healthy reference values by 7.7–26.6%, depending on each parameter. A case-control study is needed to clarify whether the deviating parameters are due to a secondary cause, such as the increased consumption of alcohol, as reported as in a study by Dalton et al. on patients with severe courses of autochthonous HEV infection [31]. Nevertheless, ALT activity was significantly increased in donors positive for anti-HEV IgG compared to those negative for anti-HEV IgG, potentially indicating that influences on ALT levels become more visible in the late stage of HEV infection. Similar findings have been observed in a Chinese study of HEV-4 infected blood donors [32]. By contrast, we found no direct correlation of viral load with ALT activity, whereas a lower viral load was associated with the detection of anti-HEV IgM and anti-HEV IgG antibodies, indicating a successful clearance of the virus by the adaptive immune system.

One limitation of our study was a drop in prevalence when the sensitivity of the measurement method was changed. The cause of this can only be determined by a comparative study on both systems used. A certain number of HEV infections with a low viral load may have been overlooked due to the routine testing in an MP of 96 samples, and thus the prevalence could have been underestimated.

Furthermore, considerations on the origin of infection and secondary influencing factors might be clarified in a subsequent case control study.

## Conclusions

This study is the first to present data on HEV infections in a large blood donor population over a period of several years in Germany and thus provides unique insight into the epidemiology of this infectious disease. Furthermore, our results show a correlation between the course of infection (serostatus and viral load) and liver parameters such as ALT, offering an interesting starting point for future studies on clinical aspects of hepatitis E in asymptomatic individuals.

## Supplementary Data

962000 Supplement
