# Supplementary material for: Hepatitis E virus infections in German blood donors: results of 8 years of screening, 2015 to 2022
Source: Euro Surveill. 2024 Jun 13;29(24):2300665. doi: 10.2807/1560-7917.ES.2024.29.24.2300665 (PMC11177570; doi:10.2807/1560-7917.ES.2024.29.24.2300665)
Supplement: Supplement [file 23-00665_PLUMERS_Supplement.pdf]

Supplementary Material

This supplementary material is hosted by *Eurosurveillance* as supporting information alongside the article 'Hepatitis E virus infections in German blood donors: results of 8 years of screening, 2015 to 2022', on behalf of the authors, who remain responsible for the accuracy and appropriateness of the content. The same standards for ethics, copyright, attributions and permissions as for the article apply. Supplements are not edited by *Eurosurveillance* and the journal is not responsible for the maintenance of any links or email addresses provided therein.

Results

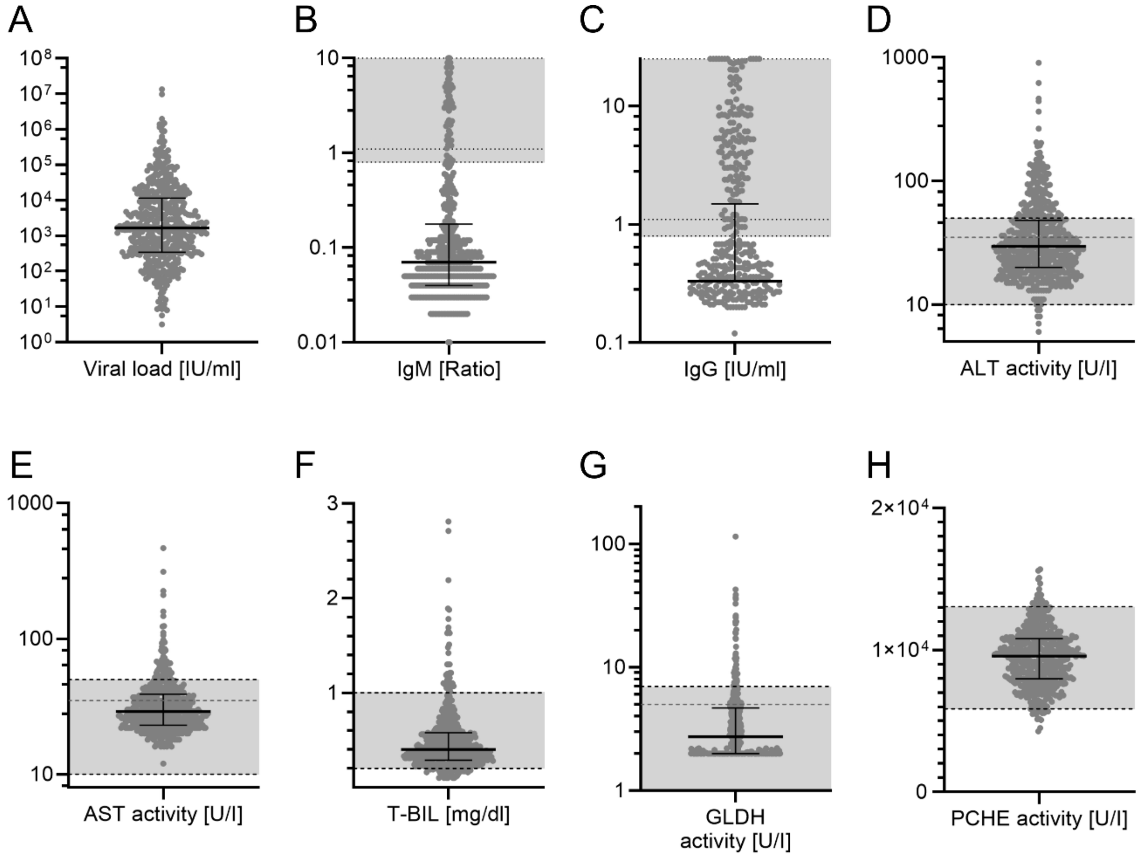

Fig. 1: Results of measurement of liver-specific parameters, serological testing and quantification of the viral load.

Displayed are the values for viral load, anti-HEV IgM and IgG antibody levels (cut-off values), alanine aminotransferase (ALT, reference range <50 U/L), aspartate aminotransferase (AST, reference range <50 U/L), total bilirubin (T-BIL, reference range 0.2-10 mg/dL), glutamate dehydrogenase (GLDH, reference range <7 U/L) and pseudocholinesterase (PCHE, reference range 5,859-13,060 U/L). The solid horizontal lines indicate median values, the dotted horizontal lines represent the cut-off values (IgM/IgG) or the reference range. The ranges defined are highlighted in grey.

The median values of the parameters measured were as followed: (a) viral load: 1,660 IU/ml (S Fig1, A), (b) anti-HEV IgM: 3.22 (S Fig. 1, B) (c) anti-HEV IgG: 4.98 (S Fig. 1, C) (b) ALT: 29.5 U/L (S Fig. 1, D), (c) AST: 29.0 U/L (S Fig. 1, E), (d) T-BIL: 0.40 mg/dL (S Fig. 1, F), (e) GLDH: 2.7 U/L (S Fig. 1, G), (f) PCHE: 9,575 U/L (S Fig. 1, H).

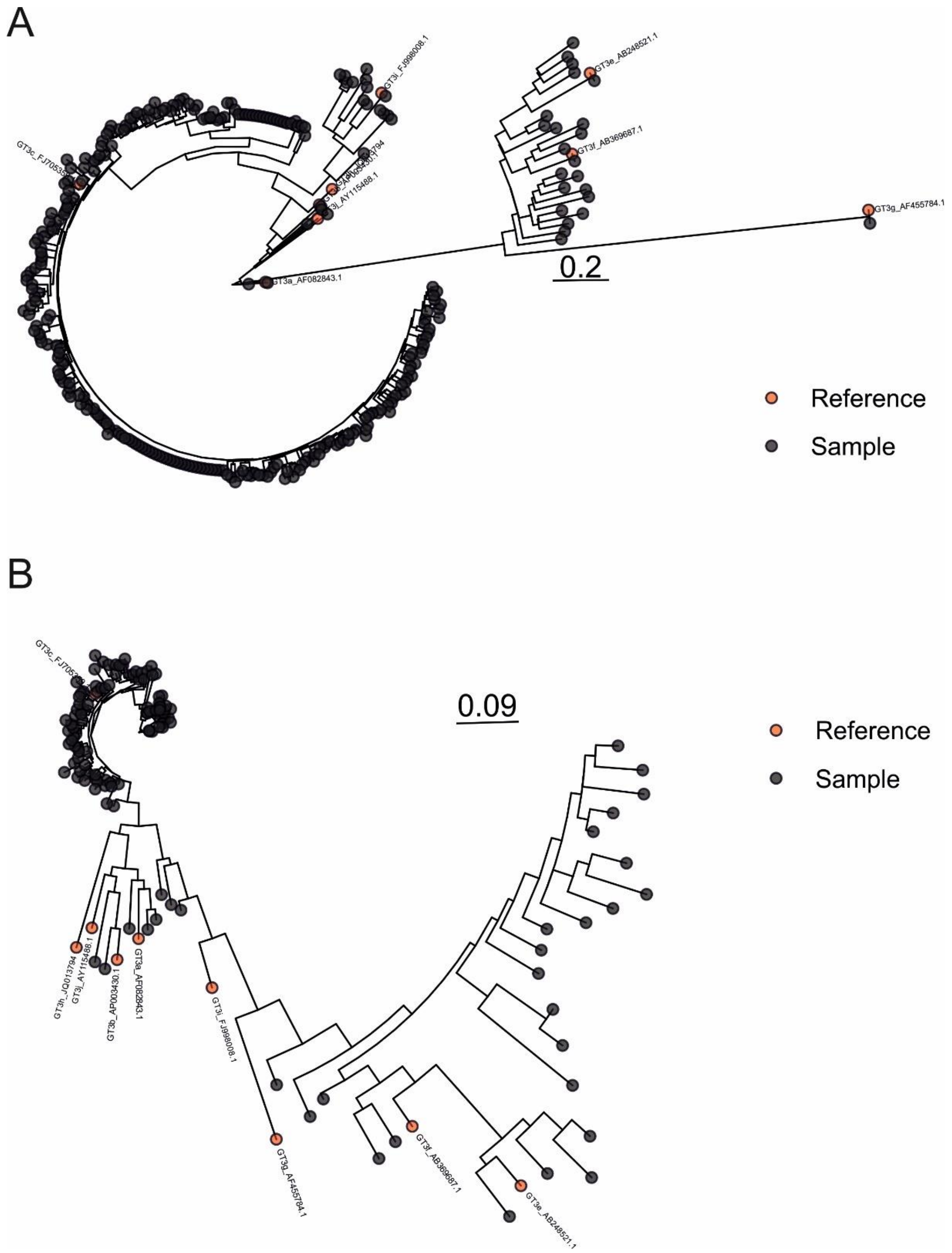

Fig. 2: Phylogenetic trees of HEV variants identified in German blood donors by sequencing data analysis in (A) the HVR and (B) the ORF1 region.

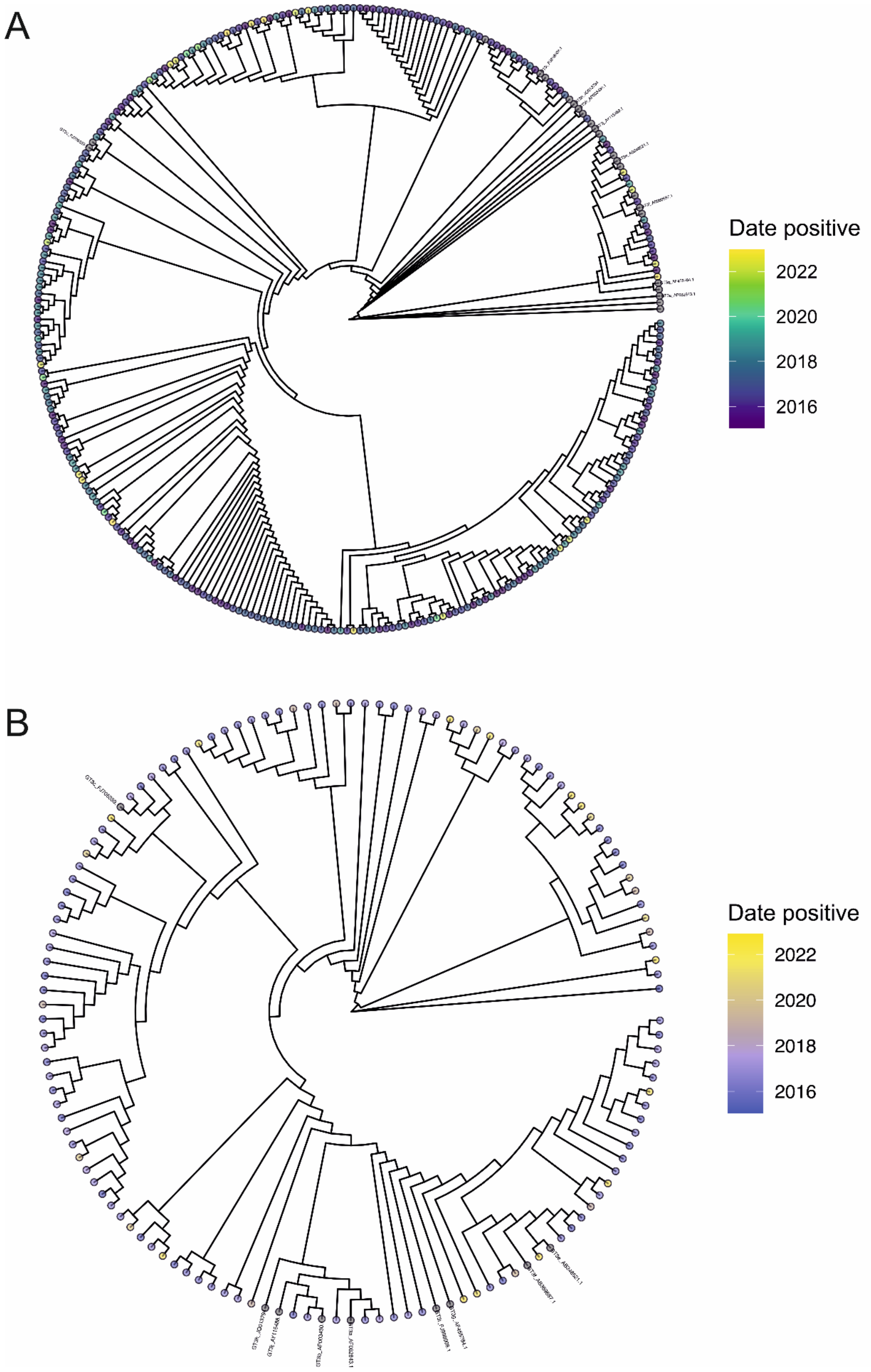

Fig.3: Phylogenetic trees of HEV variants identified in German blood donors by sequencing data analysis in (A) the HVR and (B) the ORF1 region regarding the date of donation.

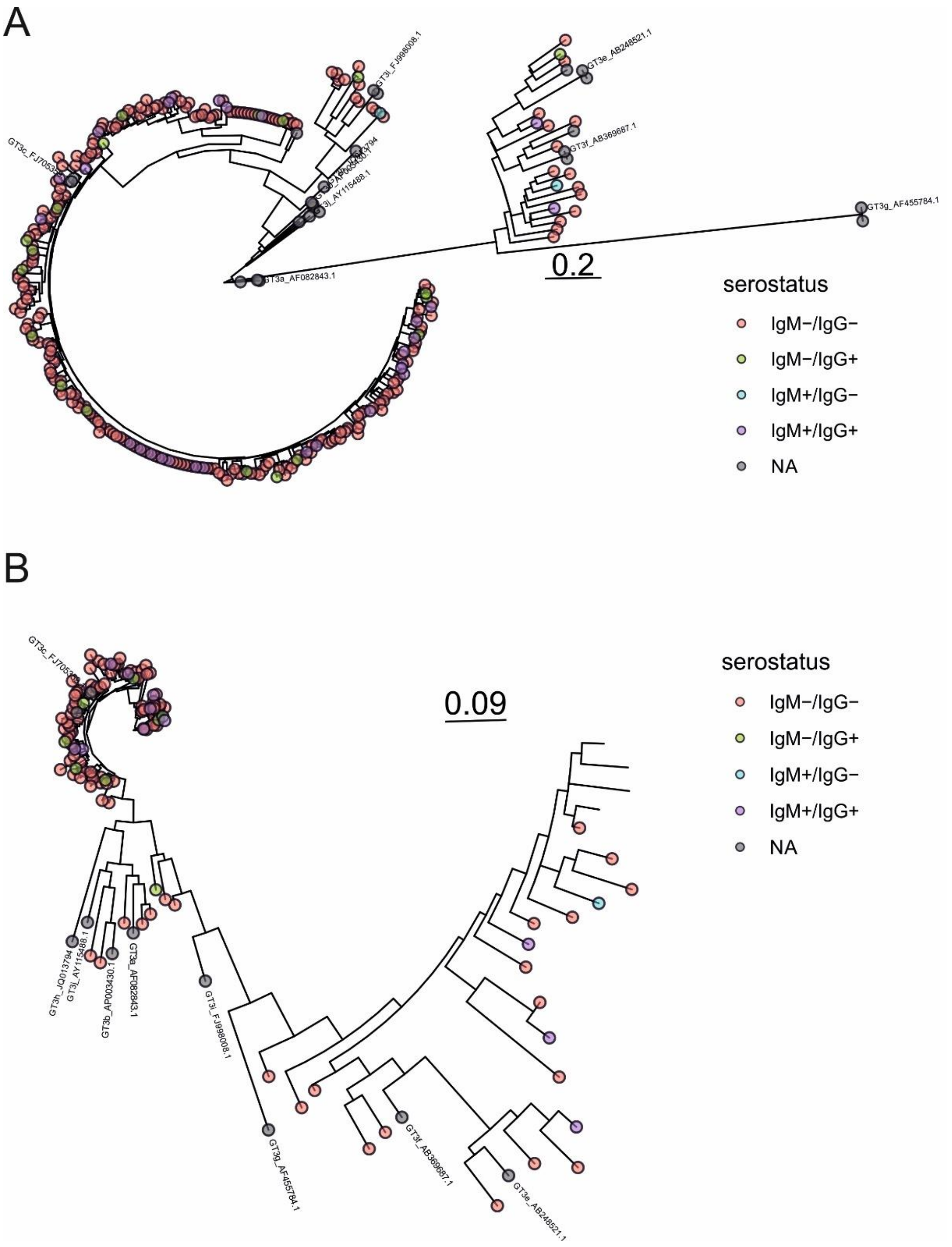

Fig.4: Phylogenetic trees of HEV variants identified in German blood donors by sequencing data analysis in (A) the HVR and (B) the ORF1 region regarding the serostatus of the donor.

Analyzed sequences were published in GenBank Database under the accession numbers PP504956 to PP505356.

Material and Methods

M1: Amplification of HEV for phylogenetic analysis

The HEV RNA was amplified by a modified nested reverse transcription PCR in the HVR and ORF1 region using primers according to Vina-Rodriguez *et al.* [13].

First round HVR: outer forward primer HEV.HVR_F1: TTYTCYCCTGGGCAYMTYTGGGA, outer reverse primer HEV.HVR_R1: TTAACCARCCARTCACARTCYGAYTCAAA (PCR product length: 401 bp).

First round ORF1: outer forward primer HEV.ORF1_F1: CCCAYCAGTTYATWAAGGCTCCTGGC, outer reverse primer HEV.ORF1_R1: TGCARDGARTANARRGCNAYNCCNGTCTC (PCR product length: 493 bp).

The first round RT-PCR amplification was performed using the Superscript III One-Step Platinum Taq RT-PCR kit (Invitrogen, Carlsbad, California, USA) in 50 µL reaction volume with 5 µL RNA eluate added to the mastermix. The final concentration of each primer was 0.4 µM for both targets (HVR and ORF1). The reverse transcription (RT) was carried out at 50 °C for 30 min, followed by denaturation/activation at 95 °C for 5 min followed by amplification of DNA with 45 cycles at 95 °C (20 s), 55 °C (45 s) and 72 °C (45 s).

Second round HVR: inner forward primers HEV.HVR_F2a: ACYTGGTCHACATCTGGYTTYT<u>C</u> and HEV.HVR_F2b: TTYTCCCCYCCTGAGGCGG<u>C</u>, inner reverse primer HEV.HVR_R2: <u>T</u>ACACCTTRGCSCCRTCRGGRTA (PCR product length: 263 or 293 bp).

Second round ORF1: inner forward HEV.ORF1_F2 AAYTCYGCCYTGGCGAATGCTGTGGTGGT, inner reverse primer HEV.ORF1_R2: CCVCGRGTNGGRGCRGWRTACCA (PCR product length: 302 bp).

Underlined bases represent trimming sites of the consensus sequence for phylogenetic analysis. The second round nested amplification for HVR was performed using the Superscript III One-Step Platinum Taq RT-PCR kit (Invitrogen) in 50 μL reaction volume with 5 μL RNA eluate added to the mastermix. The final concentration of each primer was 0.25 μM for both targets (HVR and ORF1). Initial denaturation was carried out at 95 °C for 2 min, followed by amplification of DNA with 45 cycles at 95 °C (20 s), 55 °C (45 s) and 72 °C (45 s).
